# Cellular miR-130b inhibits replication of porcine reproductive and respiratory syndrome virus *in vitro* and *in vivo*

**DOI:** 10.1038/srep17010

**Published:** 2015-11-19

**Authors:** Liwei Li, Fei Gao, Yifeng Jiang, Lingxue Yu, Yanjun Zhou, Hao Zheng, Wu Tong, Shen Yang, Tianqi Xia, Zehui Qu, Guangzhi Tong

**Affiliations:** 1 Shanghai Veterinary Research Institute, Chinese Academy of Agricultural Sciences, Shanghai 200241, P.R. China; 2 Jiangsu Co-innovation Center for Prevention and Control of Important Animal Infectious Diseases and Zoonoses, Yangzhou, 225009, P.R. China

## Abstract

MicroRNAs (miRNAs) can impact viral infections by binding to sequences with partial complementarity on viral RNA transcripts, usually resulting in the repression of virus replication. In the present study, we identified a potential binding site for miR-130 in the 5′ untranslated region (bps 155-162) of the porcine reproductive and respiratory syndrome virus (PRRSV) genome. We found that the delivery of multiple miR-130 family mimics, especially miR-130b, resulted in inhibition of PRRSV replication *in vitro*. miR-130 was effective in inhibiting the replication of multiple type 2 PRRSV strains, but not against vSHE, a classical type 1 strain. miR-130 over-expression did not induce IFN-α or TNF-α expression in either uninfected or PRRSV-infected porcine alveolar macrophages. Results from luciferase reporter assays indicated that miR-130 directly targeted the PRRSV 5′ UTR. Intranasal inoculation of piglets with miR-130b exhibited antiviral activity *in vivo* and partially protected piglets from an otherwise lethal challenge with HP-PRRSV strain vJX143. Overall, these results demonstrate the importance of the miR-130 family in modulating PRRSV replication and also provide a scientific basis for using cellular miRNAs in anti-PRRSV therapies.

Porcine reproductive and respiratory syndrome (PRRS) causes late term abortions and respiratory disease, particularly in young pigs^1^. Porcine reproductive and respiratory syndrome virus (PRRSV), the causative agent of PRRS, causes persistent infection and immunosuppression^2^. PRRSV is one the most economically important viral pathogens in pigs and the significant economic losses to the swine industry have stimulated searches for new ways to control PRRSV transmission.

MicroRNAs (miRNAs) are endogenous, noncoding, small RNAs that function as gene regulators, most commonly by mediating translational repression or degradation of target mRNAs^3^. The seed region (2–8 nucleotides at the 5′ end) of an miRNA is commonly considered as the key to exerting its silencing function, most often by binding to the 5′ or 3′ untranslated region (5′- or 3′-UTR) of an mRNA sequence^4^,^5^. There is a growing body of evidence that cellular miRNA-mediated RNAi plays a significant role in the intricate networks of host-virus interactions^6^,^7^,^8^,^9^,^10^. For example, miR-122, a liver-specific miRNA, facilitates hepatitis C virus (HCV) replication by binding to the 5′ UTR of the viral genomic RNA^11^,^12^. Let-7c inhibits H1N1 influenza A virus replication by directly targeting the 3′ UTR of viral gene M1 ( + ) cRNA^13^.

PRRSV has been characterized into two distinct genotypes^14^,^15^. The 5′ UTR of type 1 and type 2 PRRSV have different lengths (approximately 220 and 190 nucleotides (nt), respectively) and share approximately 60% nucleotide homology^16^,^17^. Several recent studies suggest a role for miRNA-mediated gene regulation in PRRSV pathogenesis^18^,^19^,^20^,^21^,^22^,^23^,^24^. Our previous study identified that mir-26a could suppress PRRSV replication by activating the type I interferon pathway^23^. Here we sought to identify additional antiviral miRNAs by computational analysis of the PRRSV genome. We found that miR-130 family members directly target the 5′ UTR of the PRRSV genome and inhibit viral replication both *in vitro* and *in vivo*. Over-expression of miR-130b inhibited multiple type 2 strains in a dose-dependent manner, but had no impact on the replication of type 1 strains. Our study reveals an example of an miRNA that modulates PRRSV replication and also highlights a host factor that could be used for RNAi-mediated antiviral therapeutic strategies.

## Results

### MiR-130 family members inhibit PRRSV replication

**Figure 1:**
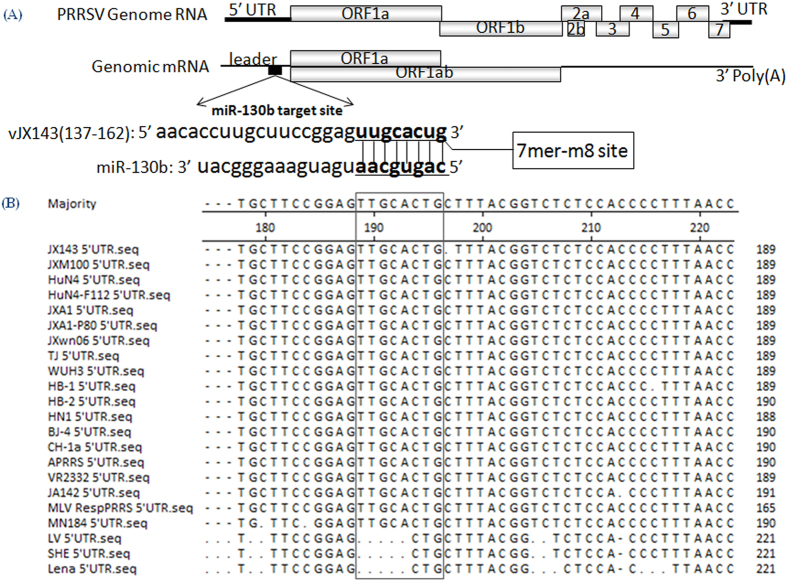
Computational prediction of potential miR-130 target sites in PRRSV genomic RNA. (**A**) Depiction of the PRRSV genomic RNA and potential mIR-130 binding sites. (**B**) Sequence alignments of 21 representative type 2 PRRSV strains and 3 type 1 PRRSV strains.

We used ViTa^25^ to predict miRNA target sites in the genomes of the HP-PRRSV strain vJX143 and the classical PRRSV strain vAPRRS. The results indicated that miR-130 might target bps 155 to 162 in the viral genomic RNA through seed base pairing (Fig. 1A). We aligned the target sequences in 24 representative PRRSV strains, covering two genotype and different virulence. The target region was 100% conserved in 21 type 2 strains, which now circulate in most commercial swine industries throughout the world, but was absent in 3 type 1 strains (Fig. 1B). Since all members of the same miRNA family^4^,^26^ (i.e., miRNAs with the same sequence at nucleotides 2 to 8) share the same predicted targets, we synthesized all the five members of miR-130 family, including miR-130a, miR-130b, miR-301a, miR-301b, and miR-454 (Table 1) based on the mature miRNA sequences annotated in miRBase (http://www.mirbase.org/)^27^. The first nucleotide at the 5′ end of miR-130 family members, except for miR-454, is C (Table 1) and the C is also complementary to the G in the viral genome (taking miR-130b as an example) (Fig. 1A), suggesting that the binding site length of miR-454 is one nucleotide shorter than that of other members.

**Figure 2:**
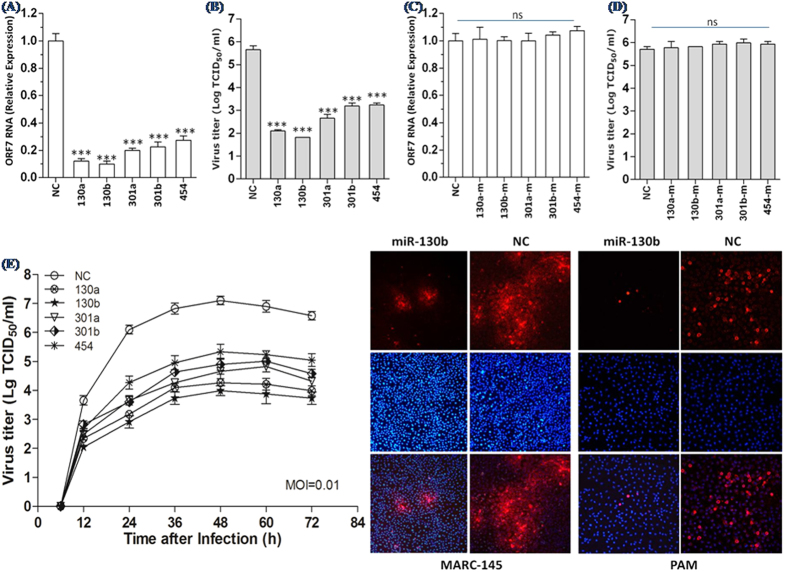
MiR-130 family members inhibit PRRSV replication. ORF7 mRNA expression level (**A,B**) and viral titers (**C,D**) in MARC-145 cells transfected with the indicated miRNAs mimics or mutants; NC = negative control mimic for 24 h prior to PRRSV strain vJX143 infection at a multiplicity of infection (MOI) of 0.01 for 36 h. Virus titers were expressed as the lg TCID_50_/ml. (**E**) PRRSV growth in MARC-145 cells transfected with miR-130 family mimics. Culture supernatants were collected at the indicated times and titrated. (**F**) Immunofluorescence staining against the PRRSV N protein after transfection and PRRSV vJX143 infection. MARC-145 cells or PAMs were fixed at 36 h post-infection and immunostained with the mouse monoclonal SDOW17 antibody against the viral N protein and FITC-conjugated goat anti mouse IgG. Cellular nuclei were counterstained with DAPI (1 mg/ml). Data are the mean + standard deviation of three independent experiments. Statistical significance was analyzed using t-tests; *P < 0.05; **P < 0.01; ***P < 0.001; ns, not significant.

We transfected chemically modified miR-130 mimics and mutants (80 nM miRNA) into MARC-145 cells, infected these cells with PRRSV vJX143 at an MOI of 0.01, and then examined virus production and viral gene expression by using virus titer assays and quantitative real-time PCR (qRT-PCR). MARC-145 cells transfected with miR-130 mimics yielded significantly lower PRRSV titers and ORF7 gene expression, as compared with cells transfected with the NC mimic (Fig. 2A,B) at 36 h post-infection. Transfection of miR-130 mutants had no significant impact on ORF7 RNA levels and virus titers in MARC-145 cells (Fig. 2C,D), indicating that the miR-130 family has antiviral activity against PRRSV. miR-130a/b seemed to be more efficient suppressors than miR-301a/b (Fig. 2A–D).

Since the miR-130 family is highly conserved between monkeys and pigs, we conducted the subsequent investigations in PAMs, which are the target cells of PRRSV infection *in vivo*. We analyzed the growth dynamics of HP-PRRSV isolate vJX143 in PAMs transfected with miR-130 family or NC mimics and found that miR-130b was the most efficient at suppressing viral growth, as compared with the other four members (Fig. 2E). Viral growth was suppressed about 1,000-fold in PAMs transfected with miR-130b during PRRSV infection after 72 h and about 100-fold in PAMs transfected with miR-454. These data were corroborated using an immunofluorescence assay in which we found that transfecting miR-130b reduced N protein expression in both PAMs and MARC-145 cells (Fig. 2F).

### MiR-130b inhibits multiple type 2 strains in a dose-dependent manner, but does not inhibit a type 1 strain

**Figure 3:**
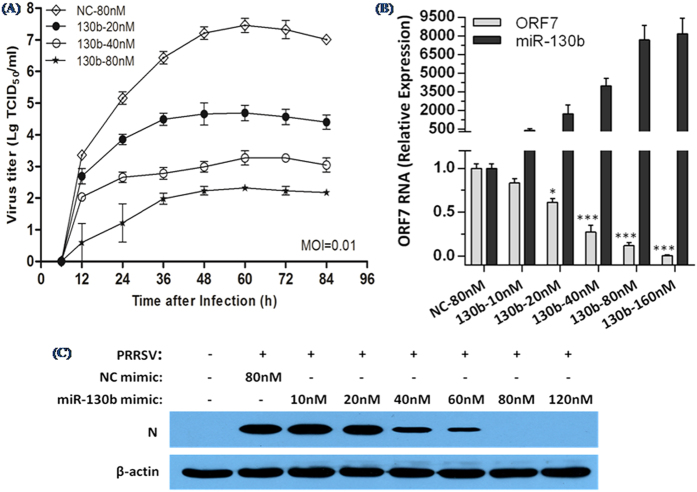
Overexpression of miR-130b reduces replication of PRRSV in a dose-dependent manner. (**A**) vAPRRS growth in MARC-145 cells transfected with NC or miR-130b mimics at the indicated doses (20, 40, 80 nM). Culture supernatants were collected at the indicated times and titrated. (**B**) qRT-PCR analysis of ORF7 RNA and miR-130b expression levels in MARC-145 cells transfected with miR-130b or NC mimics at the indicated doses (10–160 nM), followed by vAPRRS infection. The data were normalized to β-actin or U6 expression. Statistical significance was analyzed using t-tests; *P < 0.05; **P < 0.01; ***P < 0.001. (**C**) The experiments were performed as described for panel B, except that the indicated doses (10–120 nM) were used. Cells were collected at 48 h post-infection for western blot analysis of N protein expression. β-actin expression was analyzed as a loading control.

To corroborate our findings above with miR-130b further, MARC-145 cells were transfected with increasing concentrations of miR-130b mimics (20, 40, 80 nM) and then infected with vAPRRS. Both PRRSV growth and ORF7 mRNA levels were inhibited as a function of the dose of miR-130b mimic (Fig. 3A,B). Consistent with this, transfecting the miR-130b mimic also reduced the accumulation of the PRRSV nucleocapsid (N) protein in a dose-dependent manner (Fig. 3C). To exclude the possibility that reduced PRRSV replication was due to potential toxicity of the miR-130b mimic, MARC-145 cells were transfected with the miR-130b mimic at different doses (40 nM, 80 nM, and 160 nM). No appreciable effect of the miR-130b mimic on cellular viability or morphology was observed (data not shown).

**Figure 4:**
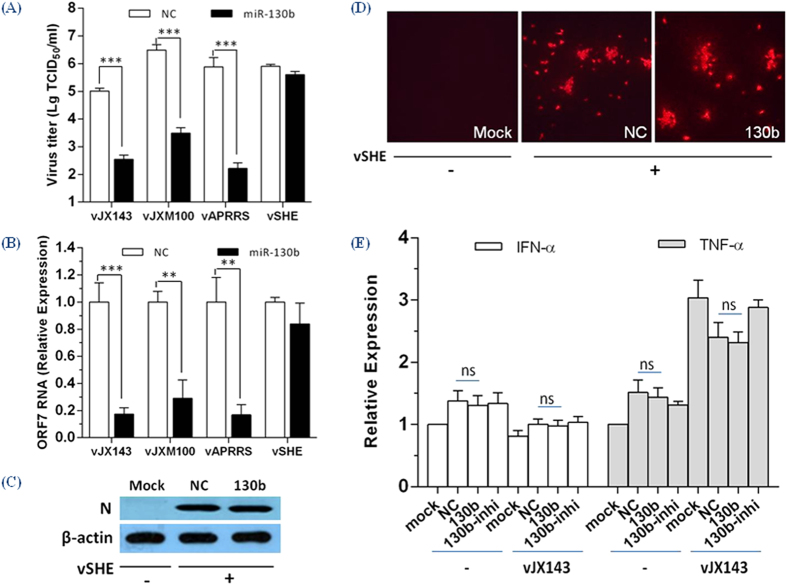
Over-expression of miR-130b inhibits multiple type 2 PRRSV strains but not a type 1 strain. (**A**) Viral titers and **(B**) ORF7 mRNA expression in MARC-145 cells transfected with NC or miR-130b mimics (80 nM) for 24 h prior to PRRSV (vJX143, vJXM100, vAPRRS or vSHE) infection (MOI = 0.01) Data are presented as mean ± SD. Statistical significance was analyzed using t-tests; **P < 0.01; ***P < 0.001; ns, not significant. (**C**) MARC-145 cells were transfected with NC or miR-130b mimics for 24 h, infected with vSHE for 48 h and collected for western blot analysis of N protein expression. β-actin expression was analyzed as a loading control. (**D**) Immunofluorescence staining against vSHE N protein as described for panel (**C**), except that cells were fixed at 24 h post-infection. (**E**) qRT-PCR analysis of IFN-α and TNF-α expression in PAMs transfected with NC, miR-130b mimics or inhibitors, and then infected with vJX143 for 36 h at an MOI of 0.01 or left untreated. Data were normalized to GAPDH expression. Statistical significance was analyzed using t-tests; ns, not significant.

The potential target region of miR-130b is 100% conserved in 21 type 2 strains, but is absent in 3 type 1 strains. We further investigated the antiviral activity of miR-130b against three other type 2 PRRSV (vAPRRS, vJX143, vJXM100) strains and a classical type 1 PRRSV strain (vSHE) in MARC-145 cells. Over-expression of the miR-130b mimic reduced virus titers and ORF7 gene expression in the 3 type 2 PRRSV strains, but not in vSHE (Fig. 4A,B). miR-130b had no impact on N protein expression from vSHE (Fig. 4C,D).

### MiR-130b does not affect IFN-α and TNF-α mRNA expression in PAMs

Two previous studies implicated miR-130 family members in regulating the innate immune system. Zhang *et al.* found that miR-130 modulated the NF-κB pathway by targeting TNF-α in cervical cancer cells^28^. Li *et al.* showed that miR-130 could upregulate type I IFN and decrease the expression of miR-122 in Huh-7.5.1 cells^29^. To determine whether miR-130 performed a similar role in PAMs, we analyzed IFN-α and TNF-α expression levels in PAMs transfected with miR-130b mimics or inhibitors. Over-expression of miR-130b failed to increase IFN-α or TNF-α mRNA expression in mock or PRRSV-infected PAMs (Fig. 4E), suggesting that miR-130 does not induce the innate immune response in PAMs.

### MiR-130 family members directly target the PRRSV genome

**Figure 5:**
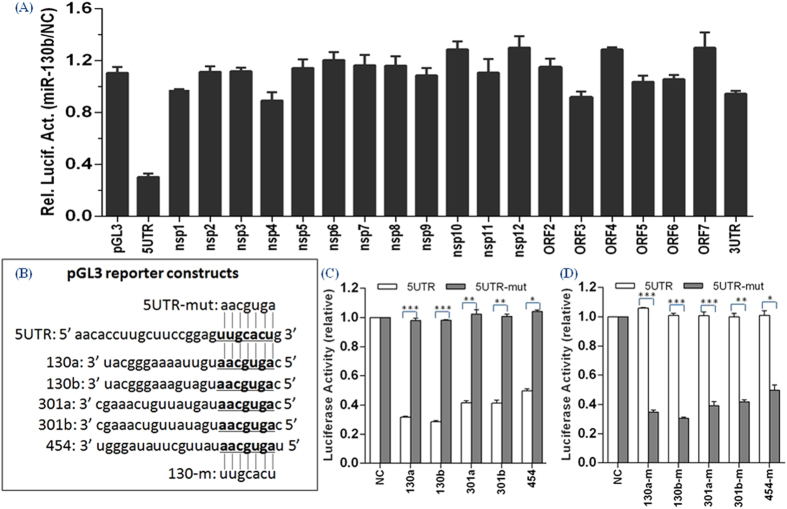
MiR-130 directly targets the PRRSV 5′ UTR. (**A**) BHK-21 cells were co-transfected with miR-130b or NC mimics and the indicated luciferase reporters. At 24 h post-transfection, cells were lysed for luciferase assays. The relative luciferase activities (miR-130b/NC) refer to the fold-change in luciferase activity in cells transfected with miR-130b mimics relative to cells transfected with NC mimics. (**B**) Predicted binding region for members of the miR-130 family. Underlined nucleotides indicate nucleotides replaced with sequences indicated by the vertical lines in miR-130 family mutants or mutant reporter constructs. Luciferase activity in lysates of BHK-21 cells co-transfected with 5′ UTR or 5′ UTR-mut reporter constructs as indicated and either (**C**). miR-130 mimics (130a, 130b, 301a, 301b, 454) or (**D**) miR-130 mutants (130a-m, 130b-m, 301a-m, 301b-m, 454-m). Data are presented as mean ± SD. Statistical significance was analyzed using t-tests; *P < 0.05; **P < 0.01; ***P < 0.001.

We then determined whether the miR-130 family specifically targets the PRRSV genome to exert its antiviral effect (Fig. 1A). Among 20 different vectors containing various PRRSV cDNA fragments, only the relative luciferase activity driven by pGL3-5UTR was significantly reduced by the miR-130b mimic, as compared with cells transfected with the NC mimic (Fig. 5A). To verify further that the direct target sites (bp 155 to 162) in the viral genomic RNA were involved in the inhibition of PRRSV replication, we generated another reporter construct with mutations at positions corresponding to the miR-130 seed region (pGL3-5UTR-mut; Fig. 5B). All 5 miR-130 family members significantly inhibited the luciferase activity of pGL3-5UTR but did not suppress pGL3-5UTR-mut activity (Fig. 5C). Reciprocally, the 5 miR-130 mutants significantly inhibited the luciferase activity of pGL3-5UTR-mut but did not affect pGL3-5UTR activity (Fig. 5D). Thus, the miR-130 family directly targets the PRRSV genome.

### Intranasal delivery of miR-130b exhibits antiviral activity *in vivo*

**Figure 6:**
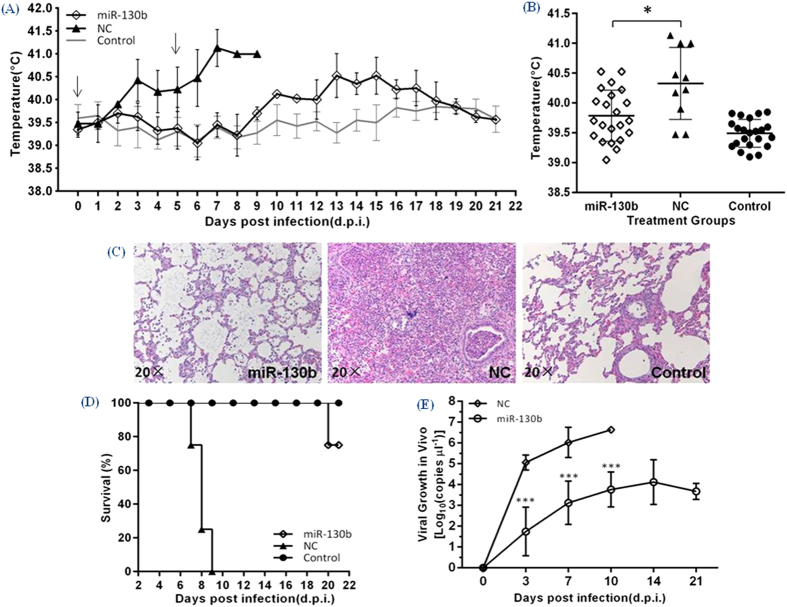
Intranasal delivery of miR-130b exhibits antiviral activity *in vivo*. (**A**) Mean rectal temperatures of piglets from two groups infected with vJX143 as well as a control uninfected group. The arrows indicate the day of NC or miR-130b administration. (**B**) Distribution of average rectal temperatures. Each data point represents an average value of rectal temperature of each group on one day throughout the 21-day period. (**C**) Histopathology analysis from the lungs and (**D**) survival curves of piglets in three groups. Log rank test; P = 0.0069. (**E**) Viral growth curves in piglets treated with miR-130b or NC mimics after vJX143 infection. Viral RNA copy numbers were determined in serum samples at the indicated times by qRT-PCR analysis. Data in panel (**E**) are presented as means ± SD. Statistical significance was analyzed using t-tests; *P < 0.05; ***P < 0.001.

Finally, to confirm whether the miR-130 family could be used in therapy, we tested the anti-PRRSV effects of miR-130b in piglets. The rectal temperature of each pig was recorded daily until 21 dpi. Pigs treated with miR-130b did not experience elevated rectal temperatures until 10 dpi, while the temperature of pigs in the NC treatment group rapidly rose to 40 °C at 3 dpi (Fig. 6A). Comparison of the mean rectal temperatures of the two groups revealed a temperature about 1 °C lower in the miR-130b treatment group, as compared with the NC treatment group (Fig. 6B). The lungs of pigs in the miR-130b treatment group showed fewer pathological changes than piglets in the NC treatment group (Fig. 6C). As a highly virulent strain, vJX143 typically causes 100% mortality by 10 dpi. However, 75% of piglets treated with miR-130b survived to 21 dpi (Fig. 6D). PRRSV RNA copy number was analyzed at 3, 7, 10, 14 and 21 dpi and was found to be about 1,000-fold lower in the miR-130b treatment group, as compared with the NC group (Fig. 6E). Thus, it appears that PRRSV replication *in vivo* can be controlled by miR-130b.

## Discussion

Given the breadth of miRNA-mediated regulation of host-virus interaction, the role of cellular miRNAs in PRRSV infection is of significant interest. In the current study, we found that miR-130 family members were strong inhibitors of PRRSV replication (Figs 1 and 2). Over-expression of miR-130b inhibited the replication of multiple type 2 PRRSV isolates in a dose-dependent manner (Figs 3 and 4). The antiviral activity of miR-130 was attributable to the direct targeting of the PRRSV 5′ UTR, rather than to a stimulation of IFN-α and TNF-α expression (Figs 4 and 5). Intranasal delivery of miR-130b exhibited antiviral activity *in vivo* and provided partial protection to piglets from an otherwise lethal challenge (Fig. 6).

Many computational and experimental approaches have shown that UTRs are common miRNA targets^4^. Previous studies reported that both miR-181 and miR-23 inhibited PRRSV replication by directly binding to the 3′ UTR of viral genomic and subgenomic RNAs^19^,^21^. We found that miR-130 instead targets the 5′ UTR of PRRSV (Fig. 1A). This region is highly conserved among type 2 PRRSV, which now circulates in most commercial swine industries through out the world^30^, but is absent in type 1 PRRSV (Fig. 1B).

The results from vJX143 growth assays in PAMs showed that miR-130a and miR-130b were more efficient at suppressing viral growth than were miR-301a, miR-301b, and miR-454 (Fig. 2E). The five miR-130 family members share the same seed region (2 ~ 8 nt at the 5′ end), but cause different inhibitory effects on PRRSV growth. We found that the first nucleotide at the 5′ end of miR-454 is different from other members (Table 1) and the binding site length of miR-454 is one nucleotide shorter, possibly accounting for the weak inhibition of PRRSV growth by miR-454.

Two previous studies implicated the miR-130 family in regulating the innate immune system by targeting TNF-α in cervical cancer cells^28^ and by triggering the type I IFN pathway in the Huh-7.5.1 cell line^29^. By contrast, we found no significant change in either IFN-α and TNF-α expression in PAM cells transfected with miR-130b mimics (Fig. 4E), suggesting the miR-130b antiviral activity is more likely attributable to its direct targeting of viral RNA (Fig. 5). We attributed one reasonable cause of this finding to cell specificity. PAM cells as normal macrophage cells showed different behavior to cancer cells against the miR-130b over-expression. Also one miRNA could commonly modulate hundreds of target genes in mammals due to the short length of binding sites, always 8–10 nucleotides^26^. MiR-130 family still might affect other target genes in PAMs which need further experiments to identify.

MiRNAs are considered to have potential efficacy as antiviral therapeutics^31^. The HP-PRRSV strain can cause high fever, high morbidity, and high mortality in pigs^30^,^32^. The intranasal inhalation delivery route has been used in many siRNA-mediated therapies against other respiratory viruses^33^,^34^,^35^. Guo *et al.* provided the only direct evidence that therapeutic miR-181c delivery could reduce the severity of infection in pigs infected with HP-PRRSV, but still cause 100% mortality^21^. In our study, intranasal delivery of miR-130b exhibited antiviral activity *in vivo* and provided partial protection to piglets from an otherwise lethal challenge of vJX143. Developing miR-130b as an antiviral therapeutic approach must be rather limited against those strains that have homology and emergence of viral resistance to the miRNA due to mutations in the target sequence is also possible. However, the animal experiments on miRNAs is opening exciting avenues for understanding host-pathogen interactions and for developing therapeutic approaches to treatment of infectious diseases, viral infections in particular.

Overall, our study demonstrates the importance of the miR-130 family in modulating PRRSV replication and also highlights the therapeutic possibility of using miRNAs to control PRRSV infections.

## Methods

### Cells and viruses

MARC-145 (ATCC, Manassas, VA) and baby hamster kidney cells (BHK-21; ATCC CCL10) were cultured as described^36^,^37^. Porcine alveolar macrophages (PAMs) were obtained by lung lavage of 4-week-old PRRSV-negative piglets as described previously^38^ and maintained at 37 °C in RPMI 1640 (Gibco) supplemented with 10% fetal bovine serum (FBS) and penicillin-streptomycin. vJX143 (GenBank accession No. EU708726) is a highly pathogenic PRRSV strain isolated from a swine farm in Jiangxi Province, China, in 2006^39^. vJXM100 (GenBank accession No.GQ475526) was obtained through 100 serial passages of vJX143 in MARC-145 cells^40^. vAPRRS (GenBank accession No. GQ330474) and vSHE (GenBank accession No. GQ461593) were rescued from the infectious cDNA clones pAPRRS and pSHE, respectively^37^,^41^. High-titer virus stocks were obtained by infecting MARC-145 cells at low multiplicities of infection (MOIs) and titrated using standard TCID_50_ assays, and then stored at −80 °C until use.

### MiRNA target prediction and conservation analysis

miRNA targets in vJX143 and vAPRRS were predicted using ViTa (http://vita.mbc.nctu.edu.tw/)^25^. For conservation analysis, we aligned the potential target sequences in 24 representative PRRSV strains, including 21 type 2 strains and 3 type 1 strains collected from GenBank (http://www.ncbi.nlm.nih.gov/GenBank), using MegAlign software^42^.

### MiRNA mimics

miR-130 family mimics (130a, 130b, 301a, 301b, 454) were synthesized by GenePharma (Shanghai, China) as double-stranded 2′-O-methyl-modified RNA oligonucleotides. The sense sequences are listed in Table 1. miR-130 mutants and inhibitors, corresponding seed-mutated miR-130 mimics (130a-m, 130b-m, 301a-m, 301b-m, 454-m), and miR-130b inhibitor (130b-inhi) are also listed in Table 1 (underlined letters are mutated bases). The negative-control (NC) mimic sequence was 5′-uucuccgaacgugucacgutt-3′.

### Transfection of miRNA mimic and viral multi-step growth kinetics

MiRNA or NC mimics were transfected into PAMs or MARC-145 cells at a concentration of 80 nM (except for dosage experiments) using X-tremeGENE siRNA Transfection Reagent (Roche). Twenty-four hours after transfection, cells were infected with PRRSV. For analysis of PRRSV growth, supernatants (0.1 ml/well) from cell cultures were collected at indicated time points post-infection and titrated in MARC-145 cells by standard TCID_50_ assay using the method of Reed and Muench^43^ for virus quantification.

### IFA and Western blotting

Indirect immunofluorescence assays (IFA) were performed for detection of nucleocapsid (N) protein in PRRSV infected MARC-145 cells or PAMs pre-transfected with miR-130b or NC mimics^23^. After a final wash step, cell monolayers were visualized using an Olympus inverted fluorescence microscope. To measure N protein expression in dosage experiments, MARC-145 cells were transfected with miR-130b or NC mimics prior to PRRSV infection, and 48 h later, SDS-PAGE and Western blotting assays were conducted with cell lysates^23^.

### RNA isolation and qRT-PCR

Total intracellular RNA was isolated using TRIzol reagent (Invitrogen) according to the manufacturer′s instructions. PrimeScript^TM^ 1st Strand cDNA Synthesis Kit (Takara) was used for reverse transcription. Quantitative RT-PCR (qPCR) analysis was performed using a Step-one Plus real-time PCR system (Applied Biosystems). The levels of ORF7 RNA, IFN-α and TNF-α mRNA were quantified using a SYBR Premix Ex Taq^TM^ (Takara). Glyceraldehyde-3-phosphate dehydrogenase (GAPDH) mRNA was used as an endogenous control and all primers above are listed in Table 2. For detection of the miR-130b expression, a commercial miRcute miRNA First-Strand cDNA Synthesis and a miRcute miRNA qPCR Detection kit (TIANGEN) were used for reverse transcription and measuring miRNA abundance. The ubiquitously expressed U6 small nuclear RNA (TIANGEN) was used for normalization purpose. All PCR experiments were performed in triplicate and relative expression levels were analyzed using the ΔΔCt method^44^.

### Luciferase assays

Twenty pGL3 luciferase reporter plasmids containing different cDNA fragments encompassing the PRRSV genome were cloned downstream of the luciferase ORF^23^ and used for luciferase assays to test and verify predicted miR-130 target regions in the PRRSV genome. For luciferase reporter assays, subconfluent BHK-21 cells cultured in 12-well plates were co-transfected with 500 ng/well of the indicated reporter plasmid and 100 ng/well of pRL-CMV (as an internal control to normalize transfection efficiency, Promega) along with the indicated amount of miR-130b or NC mimic. Cells were lysed 24 h later for determination of firefly luciferase activities using the Luciferase assay system (Promega). Data are presented as the relative luciferase activities in miR-130b mimic-transfected cells relative to NC mimic-transfected controls and are representative of three independent experiments. To generate a miR-130 target-mutated reporter construct (pGL3-5UTR-mut), mutations at positions corresponding to the miR-130 seed region were introduced using *PstI* restriction site and mutation PCR. Mutant plasmid (pGL3-5UTR-mut) was confirmed by DNA sequencing and the primer sequences are listed in Table 2. Luciferase activity in BHK-21 cell lysates were determined as described above except that miR-130 family mimics or mutants were cotransfected into cells with two reporter constructs (PGL3-5UTR or pGL3-5UTR-mut).

### Animal experiments with intranasal delivery of miR-130b

The animal welfare committee of the Shanghai Veterinary Research Institute approved the animal experiments. The approve ID is SYXK-2011-0116. All animal studies were carried out in accordance with the approved guidelines and blinded to remove investigator bias. Twelve 4-week-old PRRSV-free piglets were obtained and divided randomly into three groups, i.e., four piglets in each group. Each treatment group was housed individually. We administered miR-130b or NC mimic (6 mg per dose) mixed with RNAi-Mate (GenePharma) in Opti-MEM^®^ I (Invitrogen) solution in a final volume of 2.5 ml intranasally to the piglets, and inoculated intranasally with 3 ml of diluted vJX143 (1 × 105 TCID_50_) 6 h later, simulating the natural route of PRRSV infection. At 5 day post-infection (dpi), second deliveries of miR-130b or NC mimics were performed using the half dose and same route. The rectal temperature of each piglet was monitored daily until 21 dpi. Viral genomic RNA in serum samples from each piglet was isolated using a QIAprep viral RNA minikit (Qiagen) and viral load was detected at 3, 7, 10, 14, and 21 dpi using one-step RT-PCR. Specific primers for quantitative analysis of viral RNA copies and listed in Table 2.

### Statistical analysis

All experiments in figures were performed with at least three independent experiments. The appropriate statistical analyses were used and are presented in each figure legend. A P value of less than 0.05 was considered significant.

## Additional Information

**How to cite this article**: Li, L. *et al.* Cellular miR-130b inhibits replication of porcine reproductive and respiratory syndrome virus *in vitro* and *in vivo*. *Sci. Rep.* **5**, 17010; doi: 10.1038/srep17010 (2015).

## Figures and Tables

**Table 1:** Sequences of microRNA (miRNA) mimics and inhibitors used in this study.

Name	Sequence (5′−3′)
miR-130a (130a)	CAGUGCAAUGUUAAAAGGGCAU
miR-130b (130b)	CAGUGCAAUGAUGAAAGGGCAU
miR-301a (301a)	CAGUGCAAUAGUAUUGUCAAAGC
miR-301b (301b)	CAGUGCAAUGAUAUUGUCAAAGC
miR-454 (454)	UAGUGCAAUAUUGCUUAUAGGGU
miR-130a-mut (130a-m)	CUCACGUUUGUUAAAAGGGCAU
miR-130b-mut (130b-m)	CUCACGUUUGAUGAAAGGGCAU
miR-301a-mut (301a-m)	CUCACGUUUAGUAUUGUCAAAGC
miR-301b-mut (301b-m)	CUCACGUUUGAUAUUGUCAAAGC
miR-454-mut (454-m)	UUCACGUUUAUUGCUUAUAGGGU
miR-130b inhibitor (130b-inhi)	AUGCCCUUUCAUCAUUGCACUG
NC	UUCUCCGAACGUGUCACGUTT
NC inhibitor (NC-inhi)	CAGUACUUUUGUGUAGUACAA

**Table 2:** Sequence of oligonucleotide primers used in this study.

Primer	Sequence (5′−3′)
ORF7-F	CCCTAGTGAGCGGCAATTGT
ORF7-R	TCCAGCGCCCTGATTGAA
IFN-α-F	AGCACTGGCTGGAATGAAACCG
IFN-α-R	CTCCAGGTCATCCATCTGCCCA
TNF-α-F	ACCACGCTCTTCTGCCTACTGC
TNF-α-R	TCCCTCGGCTTTGACATTGGCTAC
GAPDH-F	CCTTCCGTGTCCCTACTGCCAAC
GAPDH-R	GACGCCTGCTTCACCACCTTCT
JX-F	CCAGGTCTACTGCACACGATG
JX-R	TTTTCACTAGTCATTCGTGC
JX-Probe	FAM-CTCCGGTGGACGTTGCCAC-TAMRA
PGL3-5UTR-F	GCTCTAGAATGACGTATAGGTGTTGGCTC
PGL3-5UTR-mut-R	TACTGCAGGGTTAAAGGGGTGGAGAGACCGTAAAGGTCACGTTCTCCGGAAGCAAGGTGC
